# Developing a Paradigm Model for Resilience of Rural Entrepreneurial Businesses in Dealing With the COVID-19 Crisis; Application of Grounded Theory in Western of Iran

**DOI:** 10.3389/fpubh.2022.833909

**Published:** 2022-02-25

**Authors:** Yousef Mohammadifar, Nader Naderi, Ehsan Khosravi, Faranak Karamian

**Affiliations:** ^1^Department of Management and Entrepreneurship, Faculty of Social Sciences, Razi University, Kermanshah, Iran; ^2^Department of Agriculture Extension and Education, Agricultural College, Razi University, Kermanshah, Iran

**Keywords:** rural economies, business resilience, rural entrepreneurial businesses, crisis, COVID-19

## Abstract

Pandemic the COVID-19 is a global threat to rural entrepreneurial businesses with an uncertain ending. Therefore, it is necessary to provide a paradigm model to reduce the negative effects of this crisis, increase the resilience of rural entrepreneurial businesses or even turn this threat into an opportunity for the development of rural entrepreneurial businesses in the long run. This study, using a qualitative approach, investigated the resilience of rural entrepreneurial businesses in dealing with the COVID-19 crisis in Kermanshah province using a paradigm model. Using purposeful and theoretical sampling, 26 cases were selected. The tools used for data collection were open questionnaires (unstructured), individual depth interviews, and taking notes. The results provide a relatively comprehensive model that consists of six basic parts: causal conditions (included economic management, health factors, human resources management, and adaptation factors), the phenomenon (included low resilience of rural entrepreneurial businesses in the face of the COVID-19 crisis), contextual conditions (including social factors; cultural factors and psychological factors), interventing conditions (included business management and legal supports), action strategies (included Planned resilient actions and Unplanned resilient actions), finally, the consequences (included adapting to crisis conditions and increasing resilience in the long run, and also lack of adaptation to crisis conditions and lack of continuity of business survival in the long run). In general, rural entrepreneurial businesses in the face of crisis must, through planned resilience measures, both increase their business resilience in the short term, as well as develop the business and gain a competitive advantage in the long run. Finally, based on the findings and in order to developing resilience in rural entrepreneurial businesses during the COVID-19 crisis, some recommendations were presented.

## Introduction

Rural areas are considered as the center of production in developing countries, including Iran, and play a key role in ensuring the independence of each country, especially in the field of food security (1). Rurals have many economic potentials and entrepreneurial opportunities that, if properly planned, can create a dynamic and diverse economy in these areas by flourishing and exploiting them (2). The high growth rate of hidden and overt unemployment in rural society compared to urban society on the one hand and the impossibility of massive investment for the development of large industries on the other hand, has forced the government to develop entrepreneurial businesses in rural communities (3).

Rural entrepreneurial businesses are one of the most important elements of rural economy that through exploitation of entrepreneurial opportunities in rural areas cause job creation and reduce unemployment, increase income and increase productivity and ultimately achieve sustainable rural development (4). In defining rural entrepreneurial businesses, three criteria can be considered: first, that they are located in a rural area and second that they offer and sell rural services and products (5), and third that Third, these businesses are considered entrepreneurial businesses when they are based on the exploitation of entrepreneurial opportunities in the rural environment (6).

Throughout history, natural and man-made disasters and crises on various scales have had adverse effects on businesses (7). In this regard, one of the crises that has recently threatened the survival of businesses and has had an unprecedented impact on them is the COVID-19 pandemic (8). The disease, caused by Severe Acute Respiratory Syndrome Coronavirus-2 (SARS-CoV-2), was discovered in late 2019 in Wuhan, China (9, 10) and on March 21, 2020 by The World Health Organization was introduced to the world as a pandemic (11).

Although COVID-19 directly threatened people's health (12); But the implementation of anti-government policies to control this pandemic had economic consequences that caused drastic changes in the economic environment at the micro and macro levels of society (13, 14); The COVID-19 pandemic has disrupted the operations of many businesses, including rural entrepreneurial businesses, due to its small size and the vulnerability of this type of business (15). Various studies show that rural businesses have lower resilience than other types of businesses in the face of COVID-19 (15–17). Since COVID-19 has had a devastating effect on rural entrepreneurial businesses, it is essential that the resilience of these businesses be developed to control and manage the negative effects and consequences of this crisis. Also, because rural areas are typically geographically isolated and have weaker human, institutional, and financial capital than urban areas; therefore, resilience is doubly important in rural businesses (2).

Resilience is the tendency of a system to maintain organizational structure and productivity, following the disruption of that system [(18): 8]. In other words, resilience creates capabilities for businesses that can survive despite adverse conditions and be on the path of return or even development (compared to before the crisis) (16).

In this regard, the effects of the COVID-19 crisis are very noticeable for rural entrepreneurial businesses in Kermanshah province (western Iran). First, because Kermanshah province has been suffering from unemployment for many years, and second, rural businesses have been largely neglected; While paying attention to this type of business and their prosperity can have a significant impact on solving the problem of unemployment in Kermanshah province. In fact, it can be said that in the context of the COVID-19 crisis, most of the policies have focused on large-scale companies and the industrial sector in urban areas while what is important is the future of microeconomic activities, especially in rural areas (19); Because rural businesses are the most vulnerable sector in times of crisis, which in case of closure not only threatens the livelihood of villagers and rural development, but also causes migration from rural to urban areas and the development of marginalization, and also disrupts urban development (15). Even in non-crisis situations, rural businesses are threatened because of their small scale (2). The current crisis will also destroy the capital that these businesses have raised over the years. Therefore, considering the importance of rural entrepreneurial businesses on the one hand and the critical situation of the outbreak of the COVID-19 in Kermanshah province on the other hand, the purpose of this study is developing a paradigm model for resilience of rural entrepreneurial businesses in dealing with the COVID-19 crisis in Kermanshah province. Therefore, considering the above-mentioned cases, the main research questions are: What are the causal factors influencing the resilience development of rural entrepreneurial businesses during the COVID-19 crisis? What are the contextual conditions affecting the resilience development of rural entrepreneurial businesses during the COVID-19 crisis? What are the interventing conditions affecting the resilience development of rural entrepreneurial businesses during the COVID-19 crisis? What are the action strategies taken to develop the resilience of rural entrepreneurial businesses during the COVID-19 crisis? What are the consequences of the action strategies adopted to develop the resilience of rural entrepreneurial businesses during the COVID-19 crisis? What is the paradigm model for resilience of rural entrepreneurial businesses in dealing with the COVID-19 crisis?

Based on the researches in the field of this study, few and relatively related researches have been done as follows. Beninger and Francis (20) stated in a study that in order to increase the resilience of businesses during the COVID-19 crisis, the integration of nine financial, physical, social, natural, human, cultural, public, political and, most importantly, health capitals is necessary. Beninger and Francis (20) states that in order to increase business resilience, all of these capitals should be used in an integrated manner in planning. Aldianto et al. (21) stated in a study that adaptation factors such as communication technologies as well as knowledge and information factors to deal with the COVID-19 crisis are effective in business resilience. Aldianto et al. (21) emphasize that in order to increase the resilience of businesses, they must constantly monitor the environment. The level of creativity and innovation in businesses should also be enhanced in order to be more sensitive to environmental changes and to provide creative and innovative responses quickly. In addition to increasing resilience, gain a competitive advantage over taking advantage of emerging environmental opportunities. Le et al. (22) stated in a study that social networks and social capital and business management play a decisive role in increasing the resilience of businesses. Le et al. (22) also stated that physical, natural, financial, human and social resources are effective in increasing the resilience of businesses. Saad et al. (23) stated in a study that human factors, social factors, economic factors, cultural factors, infrastructure factors and institutional support are effective in increasing resilience. Portuguez Castro and Gómez Zermeño (24) stated in their research that communication with relevant institutions, human and social capital, and proper management and policy-making can improve the resilience of entrepreneurial businesses in times of crisis. Portuguez Castro and Gómez Zermeño (24) stated that business managers in critical situations in relation to institutional centers need to connect with universities, other research centers and entrepreneurial ecosystems and use their creative strategies to respond to environmental change. Portuguez Castro and Gómez Zermeño (24) stated that business managers in critical situations in the field of human and social capital should train their staff and also strengthen their social networks. Portuguez Castro and Gómez Zermeño (24) stated that business managers in critical situations in the field of proper management and policy-making should have a vision for the future and business development by identifying and exploiting opportunities. Pappas and Brown (25) in a study stated that to increase resilience during the COVID-19 crisis, entrepreneurial decisions should be made for businesses based on the current situation and existing capabilities and capacities. Pappas and Brown (25) stated that business owners should be aware of environmental changes and adopt strategies commensurate with the resources of businesses to increase their resilience. Ngin et al. (26) stated in a study that short-term responses should be provided first instead of long-term systematic resilient measures, and gradually the ability of businesses to deal with these disasters should be enhanced by strengthening crisis-related infrastructure. Hanson et al. (27) in a study stated that entrepreneurial culture is one of the factors affecting the resilience of businesses. Hanson et al. (27) stated that entrepreneurial culture increases resilient responses by exploiting entrepreneurial opportunities and promoting creativity and innovation. Hiramatsu and Marshall (28) in their research state that businesses that have used catastrophic (crisis) loans have a higher degree of resilience than other businesses that have not used these loans and also more quickly to their initial pre-crisis equilibrium state. They are back and more exposed to opportunities to improve and enhance their business.

Based on the reviewed studies, it can be said that so far few empirical studies have been conducted on the resilience of rural entrepreneurial businesses during the crisis. Also, in the studies, the resilience of businesses in the face of crises such as climate change and environmental crises has been considered more, and the resilience of rural entrepreneurial businesses in the face of health crises has been neglected. Therefore, this study seeks to develop a paradigm model for resilience of rural entrepreneurial businesses in dealing with the COVID-19 crisis with a qualitative approach.

## Methodology

### Study Site

**Figure 1:**
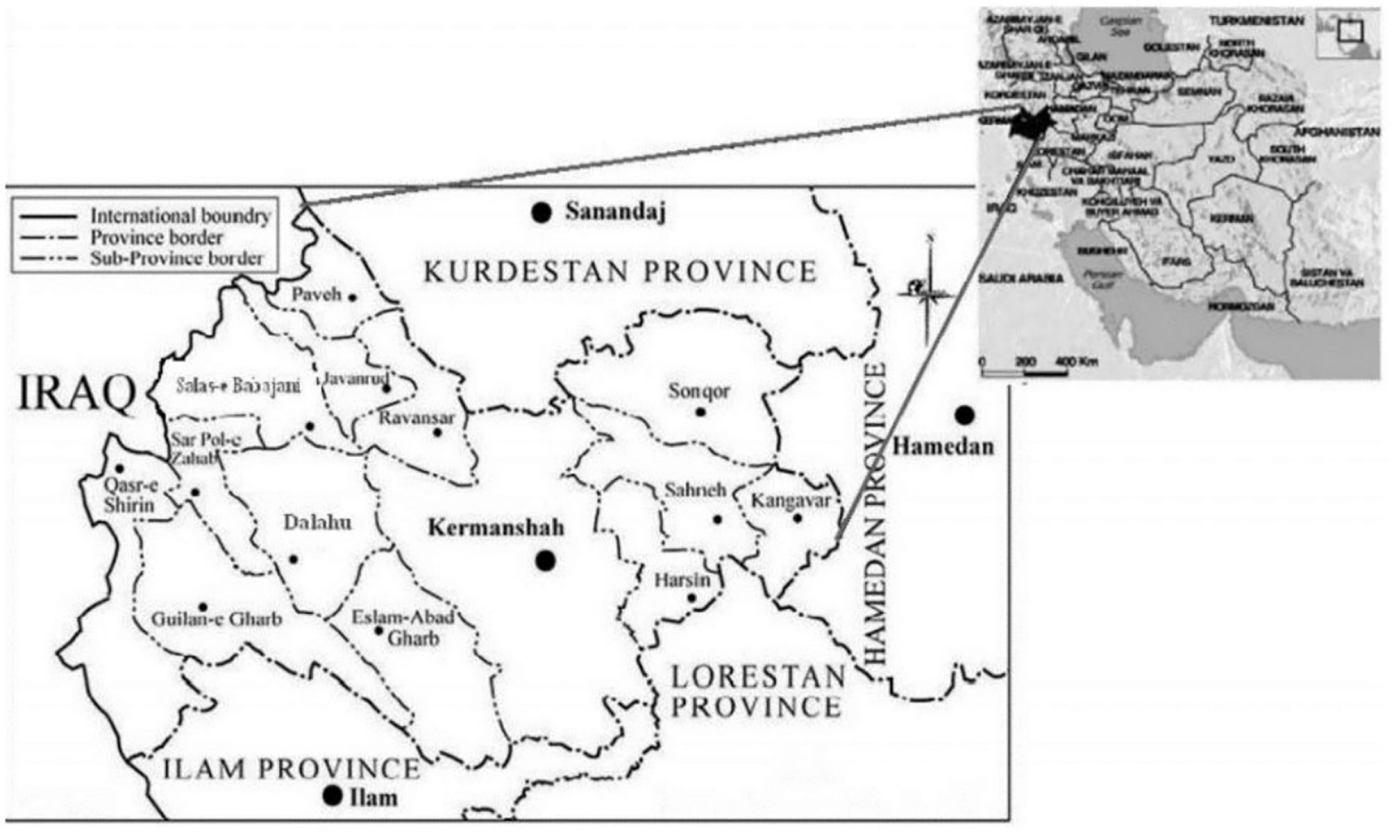
The study area; Kermanshah province, which is located in Western Iran.

The present study is limited to Kermanshah province in terms of location. The capital of Kermanshah province is the city of Kermanshah. This province has an area of 24,549 square kilometers. Kermanshah province is one of the provinces located in the west of Iran (Figure 1). Kermanshah province with an area of 24,640 square kilometers is ranked 17th among 31 provinces of Iran in terms of size and occupies 1.5% of the total area of Iran. Kermanshah province has more than 330 km of border with Iraq, this province is limited to Kurdistan province from the north, Lorestan and Ilam provinces from the south, Hamedan province from the east and Iraq from the west. According to the information of the Deputy of Statistics and Information of Kermanshah Management and Planning Organization and based on the latest divisions of the country in this province, there are 14 townships, 31 districts and 86 Counties; Figure 1; (29). Kermanshah consists of 14 districts, including Dalahu County, Gilan-e Gharb County, Harsin County, Eslamabad-e Gharb County, Javanrud County, Kangavar County, Kermanshah County, Paveh County, Qasr-e Shirin County, Ravansar County, Sahneh County, Sarpol-e Zahab County, Salas-e Babajani County, and Sonqor County (29–31) (Figure 1). The results of many researches indicate that Kermanshah province has many potentials and entrepreneurial opportunities in rural areas (32) and following the exploitation of these rural entrepreneurial opportunities, many entrepreneurial businesses have been established in rural areas of Kermanshah province (33). Various evidences show that the outbreak of COVID-19 pandemic has disrupted many rural entrepreneurial businesses in Kermanshah province (16). Therefore, it is necessary to provide a paradigm model to continue the activity and increase the resilience of rural entrepreneurial businesses in Kermanshah province during the COVID-19 crisis. Rural entrepreneurial businesses have different types that in this study, rural entrepreneurial businesses are businesses that have been established through exploitation of entrepreneurial opportunities in agricultural field in rural areas of Kermanshah province.

### Study Design

The present study seeks to provide a developing a paradigm model for resilience of rural entrepreneurial businesses in dealing with the COVID-19 crisis using the grounded theory (GT) method. GT was developed by sociologists Barney Glaser and Anselm Strauss in the mid-1960s and published in their 1967 seminal book, Discovery of Grounded Theory (34). GT is a method of extracting concepts from the heart of data and then combining them to create a theory (35). According to GT, there is no pre-determined hypothesis, but it can be achieved in the process of analysis (36). When there is no clear hypothesis, or in a region where no field research has been done so far, or little research has been done, the method of grounded theory will provide good results by creating a new theory (37). Therefore, since based on the studies, no research has been done on the research issue, especially in the study site, and also, because our goal is to arrive at a theory derived from field data extracted from the field under study, the grounded theory method gives us the best answer. Therefore, this method is most suitable for achieving the objectives of this study.

### Data Collection

Participants in this study included all experts and key informants in the field of research, such as experienced managers of rural entrepreneurial businesses in Kermanshah province who were selected through purposeful sampling and theoretical sampling. Criteria for selecting samples in this study included the following; Managers with at least 15 years of experience in rural entrepreneurial businesses, as well as managers with at least a university degree in fields related to business management, managers with a history of other business crises (with history of business management during the crisis such as the earthquake crisis in 2017 in Kermanshah province, the crisis of sanctions or the crisis of the war between Iran and Iraq and other crises). To identify the samples, purposeful sampling method was used first. In this sampling method, because the samples may not be easily identifiable at first, the researcher first identifies the key informants. In the following, the researcher reached another informed person by interviewing the informed sample and receiving the necessary data (38). In other words, after receiving information from the first key person, he was asked to introduce the person or other people who are experts in the field of research. Thus, using the initial participants, the subsequent participants were identified. In theoretical sampling based on the concept of “comparison”, data are collected based on emerging concepts (39). In this sampling, data collection is mostly done according to the categories and concepts extracted from previous data and is completed with theoretical saturation (40). Theoretical saturation occurs when no new data is discovered and no new categories are created in the open coding. In other words, theoretical saturation is a point in research where data collection seems repetitive and unproductive (41). Therefore, data collection, and consequently sample's size, continued until when new data/information no longer brought additional insights to the research questions. Therefore, theoretical saturation was obtained through interviews with 26 people. The tools used to collect the data included semi-structured interviews, field notes, and document analysis. It should be noted that each interview lasted an average of 45 to 60 min. With the permission of the participants, their voices were recorded using a tape recorder. Data collection lasted from October 1, 2021 to November 1, 2021. Because the corona virus had spread in Kermanshah province at the time of conducting research, most of the interviews were conducted in face-to-face, in accordance with health protocols and social distance, and some interviews were conducted by telephone.

The process of working in grounded theory consists of several stages. The first is to identify the research plan. The purpose of this stage is to identify the questions and constraints of the research (40, 42, 43).

The main focus of the research question is “What is this phenomenon?” (44). In this study, the general question of the research was what measures have been taken by rural entrepreneurial businesses in Kermanshah province to make their businesses resilient against COVID-19?

This study also sought to develop a paradigm model for the resilience of rural entrepreneurial businesses during the COVID-19 crisis. Therefore, the following questions were asked to the participants:

From the participants' point of view, what factors cause the development of resilience of rural entrepreneurial businesses in the face of the COVID-19 crisis? What factors affect the resilience of rural entrepreneurial businesses? What actions have been taken to develop the resilience of rural entrepreneurial businesses against COVID-19? What are the consequences of these actions?

### Data Analysis

Simultaneously with data collection, the data analysis process began. In this research, we followed Straussian grounded theory (SGT) and used a coding processor from Strauss and Corbin (44) that includes three stages: open, axial, and selective coding.

#### Open Coding

Open coding is the first data analysis process that focuses on conceptual analysis and classification of phenomena through extensive data analysis (45). In this type of coding, events are also conceptually labeled and categorized through constant comparison; In this way, concepts that have a common semantic load are placed in one category (46) and are labeled according to the semantic load of that category (34).

#### Axial Coding

**Figure 2:**
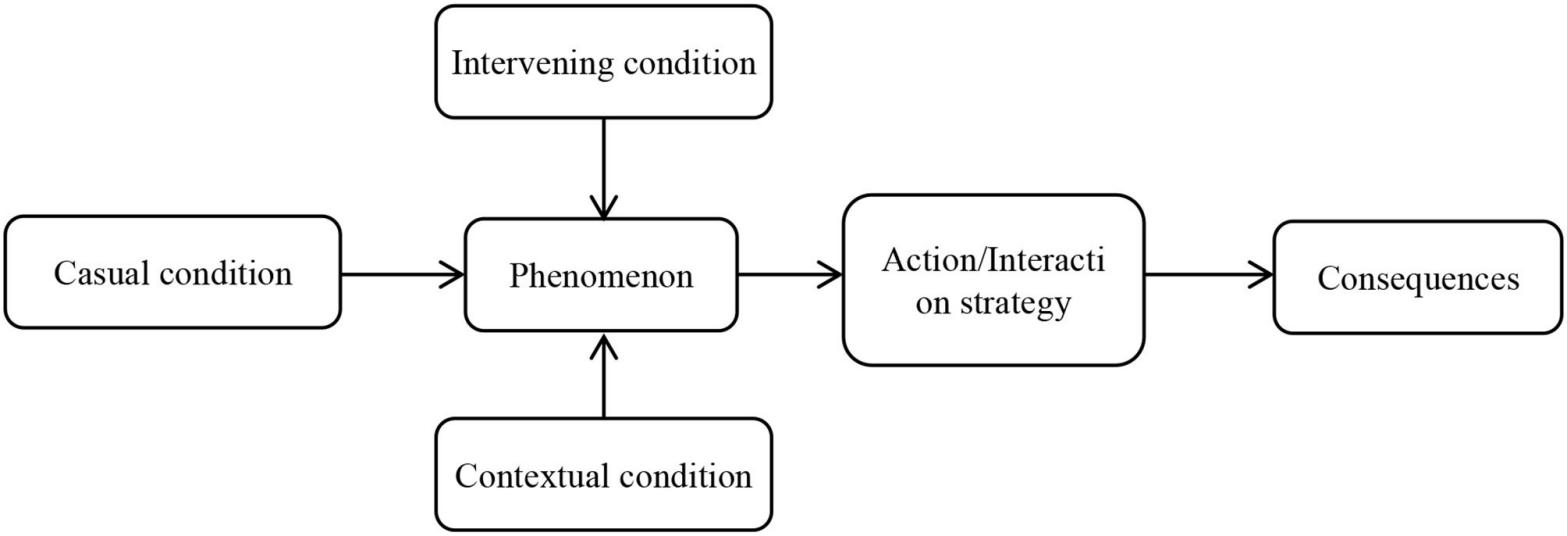
Paradigm model of Strauss and Corbin (41).

The open codes identified in the previous step are compared and clustered by axial coding, and finally they are categorized in Subcategories (39). According to Liu et al. (47) the main purpose of axial coding is to discover and establish connections between concepts and subcategories and between sub-categories and categories. In other words, axial coding means creating communication and organizing emerging communication between subcategories and achieving a comprehensive theory. Therefore, it is necessary to have a suitable design, which according to Strauss and Corbin (41), this design is the same as the paradigm model in axial coding. Through the paradigm model, casual conditions, phenomena, contextual conditions, interventing conditions, actions / strategies, and consequences are identified and subcategories are related to categories (48). As can be seen in Figure 2, the paradigm model defines six categories: causal conditions, phenomena, contextual conditions, interventing conditions, actions and consequences (41).

#### Selective Coding

While according to Strauss and Corbin (44), axial coding investigates the relationships between concepts and categories that emerge in the open coding phase, selective coding can be described as the way in which categories are linked to the main category (40). In selective coding, the main category is identified and linked to other primary categories, as well as the integration and refinement of the theory using constant comparison and storyline (47). In this research, the main category was selected and related to other categories logically and systematically through storyline writing.

### Credibility

Credibility is achieved through long-term communication and interaction with experts and data, which can be achieved through triangulation (49). For many researchers, triangulation is considered the use of data collection techniques (usually three methods) to investigate a similar phenomenon. In other words, triangulation has been interpreted as a means of cross-validating measures and validating the findings. It represents the types of data, researchers, theories, and methods (40). In this study, data triangulation was used among different types of triangulation. Typically, this process involves verifying evidence from a variety of sources to illuminate a theme or perspective (50). Data triangulation refers to the combination of different data sources that are examined at different times and places, and by different people (51). In this study, data were collected by different people (including faculty members and rural entrepreneurial business managers), at different times (from October 1, 2021 to November 1, 2021) and in different places (different villages in Kermanshah province). In other words, in this study, credibility was validated using triangulation of data sources, including participant verification, researcher debriefing and capitulations, and note-based audit sequences (52). For further confirmation, two groups, including faculty members and rural entrepreneurial business managers were employed to confirm the findings.

## Results and Discussion

### Demographic Characteristics of the Participants

73.07% (19 people) of the participants were male and the rest were female. All participants had a university degree in the field related to business management so that 65.38% (17 people) had a bachelor's degree, 26.92% (7 people) had a master's degree and 7.7% (2 people) had a PhD degree. The minimum age of participants was 34 years and the maximum age was 57 years and the average age was 46.19 years. These people also had an average experience of 25.30 years, the minimum experience was 15 years and the maximum experience was 35 years.

### Open Coding

In this research, in open coding, the data obtained from the interviews were examined line by line and the concepts were extracted. Based on the findings, 112 codes were initially identified, which in some cases were duplicates, so refinement was done and duplicate concepts were removed or expressed in the form of a concept. After the final refinement, there are 57 concepts left that were classified into different subcategories based on the semantic load. Table 1 shows the results of open coding.

**Table 1:** Open and axial coding.

**Axial coding**	**Open coding**		**References**
**Categories**	**Subcategories**	**Concepts**	
Causal conditions (A)	Aa. Economic Management	1. Receiving disaster loans (crisis);	7
		2. Income diversity;	8
		3. Previous financial resources;	4
		4. Liquidity management;	9
		5. Control and reduce costs;	6
		6. Manage and increase sales.	6
	Ab. Health factors	1. Equipping the business environment with various sanitary devices (proper ventilation, etc);	4
		2. Providing disposable health tools to personnel in order to comply with protocols (masks, etc);	9
		3. Equipping businesses with early diagnostic tools (Fever gauge, etc).	6
		4. Adherence to health protocols;	10
		5. Daily staff checkup.	8
	Ac. Human resources management	1. Continuous updating of personnel health information in dealing with the corona crisis;	8
		2. Improving the skills of personnel in observing health protocols;	10
		3. Time flexibility in the presence of personnel;	9
		4. Flexibility of location in the presence of staff (doing things remotely if possible).	9
	Ad. Adaptation factors	1. Continuous environmental monitoring, continuous planning and adaptation to environmental changes and their timely implementation;	8
		2. Provide consistent systematic responses (short, medium and long term);	7
		3. Use of crisis adaptive technologies in business model	11
Intervening conditions (B)	Ba. Business management	1. Crisis management and proper accountability;	7
		2. Condition-based planning for the business;	8
		3. Develop and present appropriate strategies for business continuity.	9
	Bb. Legal supports	1) Proper management and policy making;	12
		2) Providing infrastructure adapted to crisis situations (increasing antenna coverage in villages in remote areas);	9
		3) Support policies for low-interest (or even non-interest-bearing) lending;	9
		4) Increase partnership and cooperation between enterprises, government and other private organizations;	8
		5) Develop and provide crisis preparedness and management instructions;	8
		6) Government support in providing health services to businesses;	6
		7) Monitoring the proper implementation of health protocols.	7
Contextual conditions (C)	Ca. social factors	1) Increasing membership in indigenous and non-indigenous social networks with the aim of benefiting from experiences;	5
		2) Increasing trust, participation and cooperation in observing preventive measures among personnel and customers;	6
		3) Increase cooperation throughout the supply chain in the business.	4
	Cb. cultural factors	1) Strengthening the entrepreneurial culture in business with the aim of providing products or services that are creative and adapt to new conditions and gain a competitive advantage;	8
		2) Elimination of incorrect and opposing cultures by controlling and preventing the spread of coronavirus (elimination of handshake, etc.).	6
	Cc. psychological factors	1) Increase staff motivation in relation to business continuity;	7
		2) Increase staff resilience in relation to business continuity;	8
		3) Strengthen the motivation of personnel with coronavirus.	6
Phenomenon (D)	Da. Low resilience of rural entrepreneurial businesses in the face of the COVID-19 crisis	1) Discontinuation of rural entrepreneurial businesses in the face of the COVID-19 crisis	26
		2) Lack of adaptability of rural entrepreneurial businesses in the face of the COVID-19 crisis	25
Action strategies (E)	Ea. Planned resilient actions	1. Development and modification of marketing strategies based on crisis conditions;	10
		2. Human resource training;	6
		3. Develop and provide health instructions;	7
		4. Monitoring the proper implementation of health protocols;	8
		5. Reform of financial management based on crisis conditions;	8
		6. Development of crisis-adapted infrastructure;	8
		7. Exploiting emerging environmental opportunities.	7
	Eb. Unplanned resilient actions	1) Sales of some business equipment and machinery;	5
		2) Sale of part of the company's shares;	5
		3) Temporary deactivation of the business;	6
		4) Reducing the quantity of production;	4
		5) Decreased production quality.	5
Consequences (F)	Fa. Adapting to crisis conditions and increasing resilience in the long run	1) Business continuity;	8
		2) Recovery of business position;	8
		3) Improvement of business position than before of crisis;	9
		4) Business growth and development;	7
		5) More prepared and developed to face future crises.	6
	Fb. Lack of adaptation to crisis conditions and lack of continuity of business survival in the long run	1) More vulnerability in the long run;	7
		2) Bankruptcy and inactivity in the long run.	6

*References refer to the number of times a concept is coded and emphasized by participants (53)*.

*Findings of the study*.

### Axial Coding (Resilience Paradigm Model)

In the present study, in axial coding, causal conditions included economic management, health factors, human resources management, and adaptation factors. The phenomenon included low resilience of rural entrepreneurial businesses in the face of the COVID-19 crisis. Contextual conditions including social factors; cultural factors and psychological factors. Interventing conditions included business management and legal supports. Finally, action strategies included Planned resilient actions and Unplanned resilient actions, the consequences of which included adapting to crisis conditions and increasing resilience in the long run, and Lack of adaptation to crisis conditions and lack of continuity of business survival in the long run (More details will be provided in the lower sections).

#### Casual Conditions

According to the perspectives of rural entrepreneurs in Kermanshah province, four factors directly cause the resilience of rural entrepreneurial businesses; these factors include economic management, health factors, human resources management, and adaptation factors.

##### Economic Management

From the participants' point of view, economic management causes the resilience of rural entrepreneurial businesses during the COVID-19 crisis. Participants stated that although COVID-19 directly affects the health of human resources, it has economic consequences for rural entrepreneurial businesses due to the implementation of adaptive and preventive strategies to prevent the spread of COVID-19. Therefore, the participants believed that under these conditions, which face economic problems, the best factor that increases the resilience of rural entrepreneurial business is economic management. Therefore, rural entrepreneurs should seek to increase liquidity through various means including receiving disaster loans (crisis), income diversity, previous financial resources, liquidity management, control and reduce costs, manage and increase sales to improve the resilience of their business.

This part of the results is parallel to findings Beninger and Francis (20); Le et al. (22); Saad et al. (23) and Hiramatsu and Marshall (28). Beninger and Francis (20); Le et al. (22); Saad et al. (23) in separate studies stated that having financial factors will increase business resilience. Hiramatsu and Marshall (28) state in their research that businesses that have used catastrophic (crisis) loans have a higher degree of resilience than other businesses that have not used these loans. In explaining this part of the findings, it should be stated that one of the main consequences of various crises for businesses is economic consequences. In the meantime, those businesses have more resilience that have the ability to better manage the economy in times of crisis. In other words, in times of crisis, businesses must seek to reduce costs and increase their liquidity and revenue, and manage their financial resources to better overcome the crisis. Rural entrepreneurial businesses are usually not in a good financial position due to their small size and their financial resilience is very low, so what develops the resilience of rural entrepreneurial businesses is economic management.

##### Health Factors

From the participants' point of view, another factor that increases the resilience of rural entrepreneurial businesses is to prevent the spread of the COVID-19 virus, so to increase the resilience of rural entrepreneurial businesses, it is necessary to follow health principles and protocols. In this regard, participants believed that rural entrepreneurial businesses should be equipped to deal with the COVID-19 virus. Participants stated that addressing the following health factors could cause them to deal with the COVID-19 virus in rural entrepreneurial businesses; equipping the business environment with various sanitary devices (proper ventilation, etc), providing disposable health tools to personnel in order to comply with protocols (masks, etc), equipping businesses with early diagnostic tools (Fever gauge, etc), adherence to health protocols; daily staff checkup.

This part of the results is parallel to findings Beninger and Francis (20) and Aldianto et al. (21). Beninger and Francis (20) states that health factors in the COVID-19 crisis provide the basis for increasing business resilience, and Aldianto et al. (21) in a study emphasized the factors of knowledge and information to deal with the Corona crisis. The COVID-19 crisis is of a health nature in itself and directly targets the health of business human capital. Since the biggest asset of any business is the human capital of that business, so to increase resilience, businesses must pay attention to the health of personnel and take measures to strengthen and protect the health of personnel.

##### Human Resources Management

Participants stated that the nature of the COVID-19 crisis is such that it directly targets the human capital health of rural entrepreneurial businesses. Therefore, to increase the resilience of rural entrepreneurial businesses, factors that protect human capital should be given priority. Participants stated that personnel information on how to deal with COVID-19 should be updated first, and then human capital skills should be improved through training. Personnel should also perform their duties remotely (*via* information technology and virtually) as much as possible, and staff attendance should be shifted in time.

This part of the results is parallel to findings Beninger and Francis (20); Le et al. (22) and Saad et al. (23). As mentioned, human resources are the largest and most valuable asset of any business that any business must protect to achieve increased resilience. Because the COVID-19 crisis directly targets human resource health, businesses must adopt strategies that minimize the risk to their human resources. In other words, human resources in the face of the COVID-19 health crisis must be managed to minimize damage to these valuable human resources and increase business resilience.

##### Adaptation Factors

From the participants' point of view, crises cause environmental changes, and under these conditions, the organization or business is resilient, which can adapt to the new normal conditions. From the participants' point of view, crises cause environmental changes, and under these conditions, the organization or business is resilient, which can adapt to the new normal conditions. Participants stated that these responses should be systematic and strategic, in other words, short-term responses should be in line with the vision and development of the business in the medium and long term. Participants also stated that they should avoid responses that create consistency in the short term but provide business destruction in the long term. Participants stated that in order to increase the resilience of rural entrepreneurial businesses, their business model should be redesigned to adapt to the existing conditions. In other words, the factors that adapt to the COVID-19 crisis must be added to their business model. They stated that the factors of adaptation in the COVID-19 crisis are the elimination of face-to-face interactions and the use of IT infrastructure.

This part of the results is parallel to findings Aldianto et al. (21). Explaining this part of the findings, it should be noted that the crisis is upsetting the balance of the environment and environmental changes in businesses. Therefore, in order to increase resilience, businesses must adapt to the new normal conditions, and only in this way can they overcome the crisis. What makes adaptation to new conditions is the use of crisis-adapted tools. In the COVID-19 crisis, this tool includes information technology. In other words, the way to deal with COVID-19 is to reduce face-to-face interactions and increase virtual communication. Therefore, rural entrepreneurial businesses in order to achieve maximum adaptation to the new normal conditions and increase the resilience of their business must redesign their business model and use information technology tools in their new business model.

#### Phenomenon

The phenomenon in this study is the main issue of the research. The main issue in this study is the low resilience of rural entrepreneurial businesses in the face of the COVID-19 crisis. In other words, since the COVID-19 crisis had severely disrupted and risked rural entrepreneurial businesses, it is necessary to provide a resilience paradigm model for them. Therefore, the phenomenon studied in this study includes low resilience of rural entrepreneurial businesses in the face of the COVID-19 crisis.

#### Intervening Conditions

Intervening conditions refer to aspects that affect or modify the effects or development of a phenomenon (54). In this study, the intervention conditions include business management and Legal supports. In other words, the interventionist conditions in this study are examined at both macro and micro levels and refers to the policy-making at the macro and micro levels of society.

##### Business Management

In this study, the intervening conditions at the micro level refer to the decisions, policies and governance at the level of rural entrepreneurial businesses. Participants stated that they adopted decisions and policies such as Crisis management and proper accountability, Condition-based planning for the business, and Develop and present appropriate strategies for business continuity to increase resilience at the business management level.

This part of the results is parallel to findings Le et al. (22) and Pappas and Brown (25). Le et al. (22) stated in a study that business management plays an important role in increasing the resilience of businesses in times of crisis. Pappas and Brown (25) stated in a study that in order to increase resilience in the context of the COVID-19 crisis, entrepreneurial decisions should be made for businesses based on the current situation and existing capabilities and capacities. In explaining this part of the findings, it should be stated that the difference in the success or failure of businesses in any situation is due to the decisions and strategies that are adopted and operated by those businesses. Therefore, proper and situation-based management in times of crisis leads to increased resilience. Therefore, businesses should always monitor their environment and be sensitive to even the smallest changes and provide them with an appropriate strategy to increase their resilience and overcome crises.

##### Legal Supports

In this study, intervening conditions at the macro level refer to the government's decisions and policies to increase the resilience of rural entrepreneurial businesses. Participants stated that the government has adopted appropriate policies to continue the operation of rural entrepreneurial businesses at the village level, as follows; (1) Proper management and policy making; (2) Providing infrastructure adapted to crisis situations (increasing antenna coverage in villages in remote areas); (3) Support policies for low-interest (or even non-interest-bearing) lending; (4) Increase partnership and cooperation between enterprises, government and other private organizations; (5) Develop and provide crisis preparedness and management instructions; (6) Government support in providing health services to businesses; (7) Monitoring the proper implementation of health protocols.

“Proper management and policy making” means that the government has adopted appropriate policies in response to environmental changes to increase the resilience of rural entrepreneurial businesses, and policies have been based on environmental monitoring. Also, due to the fact that most rural areas in Kermanshah province have weak antennas, the government strengthened the antenna infrastructure in these areas. The government also provided disastrous loans to compensate companies and increase the liquidity of rural entrepreneurial businesses in order to offset some of the losses. To increase the resilience of rural entrepreneurial businesses, the government also provided contexts for increased collaboration and partnership between companies, government, and other private organizations. The government also developed guidelines for how to manage crises and adhere to health protocols, and monitored how to handle them properly.

This part of the results is parallel to findings Beninger and Francis (20), Saad et al. (23) and Portuguez Castro and Gómez Zermeño (24). Beninger and Francis (20) states that macro-level supportive policies are effective in increasing business resilience. Saad et al. (23) stated that the support of relevant institutions during the crisis is effective in increasing the resilience of businesses. Portuguez Castro and Gómez Zermeño (24) stated in their research that the level of communication with relevant institutions can improve the resilience of entrepreneurial businesses in times of crisis. Explaining this part of the findings, it should be said that the government and relevant institutions with appropriate policy-making play a key role in increasing the resilience of rural entrepreneurial businesses. The government seeks to intervene to increase the resilience of rural entrepreneurial businesses by providing financial support, such as disaster lending or the development of crisis-friendly infrastructure, such as the development of IT infrastructure in less developed villages. The relevant institutions and the government, by increasing the relationship with businesses, should be aware of their latest needs to increase resilience and try to meet their needs through the development of appropriate policies.

#### Contextual Conditions

Contextual conditions refer to where a phenomenon occurs and the conditions that allow the development of a strategy (40). In other words, contextual conditions refer to those conditions that provide the context for the occurrence of the phenomenon. In this study, contextual conditions include social factors, cultural factors and psychological factors.

##### Social Factors

Participants stated that contextual social participation has increased the resilience of rural entrepreneurial businesses. Participants stated that by joining indigenous and non-indigenous social networks and sharing their experiences of dealing with the crisis, they have provided the context for increasing the resilience of rural entrepreneurial businesses. Participants stated that increased participation and cooperation in adhering to health protocols has led to virus control and thus increased business resilience. Increased cooperation throughout the supply chain in rural entrepreneurial businesses has also reduced costs and increased revenue, and this cooperation across the supply chain has provided increased resilience.

This part of the results is parallel to findings Beninger and Francis (20); Saad et al. (23) and Le et al. (22). Beninger and Francis (20); Saad et al. (23) and Le et al. (22) in separate studies stated that social factors are effective in increasing the resilience of businesses. Explaining this part of the findings, it should be said that the COVID-19 crisis is a crisis that has affected all communities, so all communities and businesses must cooperate and participate in dealing with this crisis. Given the limited experience in dealing with health crises and the unknownness of the COVID-19 virus, all business managers should share their experiences of coping with the crisis through membership in local and non-native social networks. Increasing cooperation across the supply chain can also reduce the costs of rural entrepreneurial businesses, which increases resilience. The most important end of the COVID-19 crisis is when there is maximum cooperation and participation at the community level in compliance with health protocols. In other words, what has led to the expansion of COVID-19 is the lack of cooperation and participation of communities. Therefore, in order to increase the resilience of businesses, social factors must be strengthened.

##### Cultural Factors

In this study, cultural factors include entrepreneurial culture in business and elimination of incorrect and opposing cultures by controlling and preventing the spread of coronavirus.

The emergence of crises causes a change in the balance and status of the business environment. On the other hand, environmental changes are the source of the emergence of entrepreneurial opportunities. Participants stated that if rural entrepreneurial businesses have a strong entrepreneurial culture, they can offer creative and relevant products or services for the environment by identifying emerging opportunities and exploiting them in a timely manner. In this way, not only can they improve their resilience, but they can also gain a competitive advantage and turn the crisis into an opportunity for their business to grow and develop. This part of the results is parallel to findings Hanson et al. (27). Hanson et al. (27) in their research stated that entrepreneurial culture in businesses in critical situations is an important factor in business resilience. In explaining this part of the findings, it should be stated that crises are the source of entrepreneurial opportunities due to changes in the environment. A business that adapts faster to environmental change and more quickly identifies and exploits emerging environmental opportunities has a better competitive advantage and more resilience.

Regarding the elimination of incorrect culture in the region, the participants stated that the culture of the rural community is contrary to preventive measures and health protocols. Rural community culture needs to be reformed and revised based on health protocols to control the COVID-19 crisis to provide a context for increasing the resilience of rural entrepreneurial businesses. This part of the results is parallel to findings Beninger and Francis (20) and Saad et al. (23). Beninger and Francis (20) and Saad et al. (23) in separate studies stated that cultural factors are effective in increasing business productivity. Explaining this part of the findings, it should be said that the reason for the continuation of the COVID-19 crisis may be due to the incorrect cultures of individuals in communities. If this crisis is to be tackled, the health culture of the community must first be reformed and the cultures that endanger the health of the community must be eliminated. In this regard, proper health behaviors must be created in society.

##### Psychological Factors

In this study, psychological factors including increase staff motivation in relation to business continuity, increase staff resilience in relation to business continuity, strengthen the motivation of personnel with coronavirus.

Staff motivation should be stimulated in various ways. For example, some participants pointed out that providing incentive leave can increase people's motivation and thus increase business resilience. Some participants said that the minds of personnel should be more resilient and prepared to deal with the COVID-19 crisis. In other words, personnel must be mentally prepared in advance to deal with this crisis in order to provide a more appropriate response when faced with it. Personnel infected with COVID-19 virus should be motivated. Participants stated that the most important capital of rural entrepreneurial businesses is human capital, which should be motivated and mentally supported under any circumstances.

#### Action Strategies

Action strategies are programs that can help adapt to a phenomenon, in our context (40), the resilience of rural entrepreneurial businesses in the face of the COVID-19 crisis. In other words, action strategies are the actions that the study community takes in response to the emerging phenomenon. In this study, action strategies developed by rural entrepreneurial businesses to achieve resilience to the COVID-19 crisis include planned resilient actions and unplanned resilient actions.

##### Planned Resilient Actions

Planned resilient actions refer to those short-term resilient actions that are taken during the COVID-19 crisis but are aimed at developing and growing the business in a long-term perspective. In this research planned resilient actions include development and modification of marketing strategies based on crisis conditions, human resource training, develop and provide health instructions, monitoring the proper implementation of health protocols, reform of financial management based on crisis conditions, development of crisis-adapted infrastructure and exploiting emerging environmental opportunities. This part of the results is parallel to findings Ngin et al. (26). Ngin et al. (26) stated in a study that short-term responses should be provided first instead of long-term systematic resilient measures, and gradually the ability of businesses to deal with these disasters should be enhanced by strengthening crisis-related infrastructure. In other words, temporary responses should be in line with long-term systematic responses, and any plan to increase business resilience should be parallel to the growth and development of the business in a long-term perspective.

##### Unplanned Resilient Actions

Unplanned resilient actions refer to those measures that, although in the short run increase the resilience of rural entrepreneurial business, but in the long run lead to business bankruptcy. In this study, unplanned resilient actions include sales of some business equipment and machinery, sale of part of the company shares, temporary deactivation of the business, reducing the quantity of production and decreased production quality.

In this regard, the participants stated that resilient measures should be taken in a long-term perspective that will pave the way for business growth and development. In this regard, the participants stated that resilient measures should be taken in a long-term perspective that will pave the way for business growth and development. For example, redesigning the business model and increasing the factors that adapt to the current crisis, such as taking advantage of information technology opportunities, will increase both resilience during COVID-19 and, in the long run, the growth and development of the business.

#### Consequences

The consequences of implementing resilient behaviors, in our situation, although they increase resilience in the short run, in the long run fall into two categories: adapting to crisis conditions and increasing resilience in the long run, and Lack of adaptation to crisis conditions and lack of continuity of business survival in the long run.

What is important is that adaptive measures should be taken in a way that, while being adaptable in the short run, also provides the context for the growth and development of rural entrepreneurial business in the long run.

**Figure 3:**
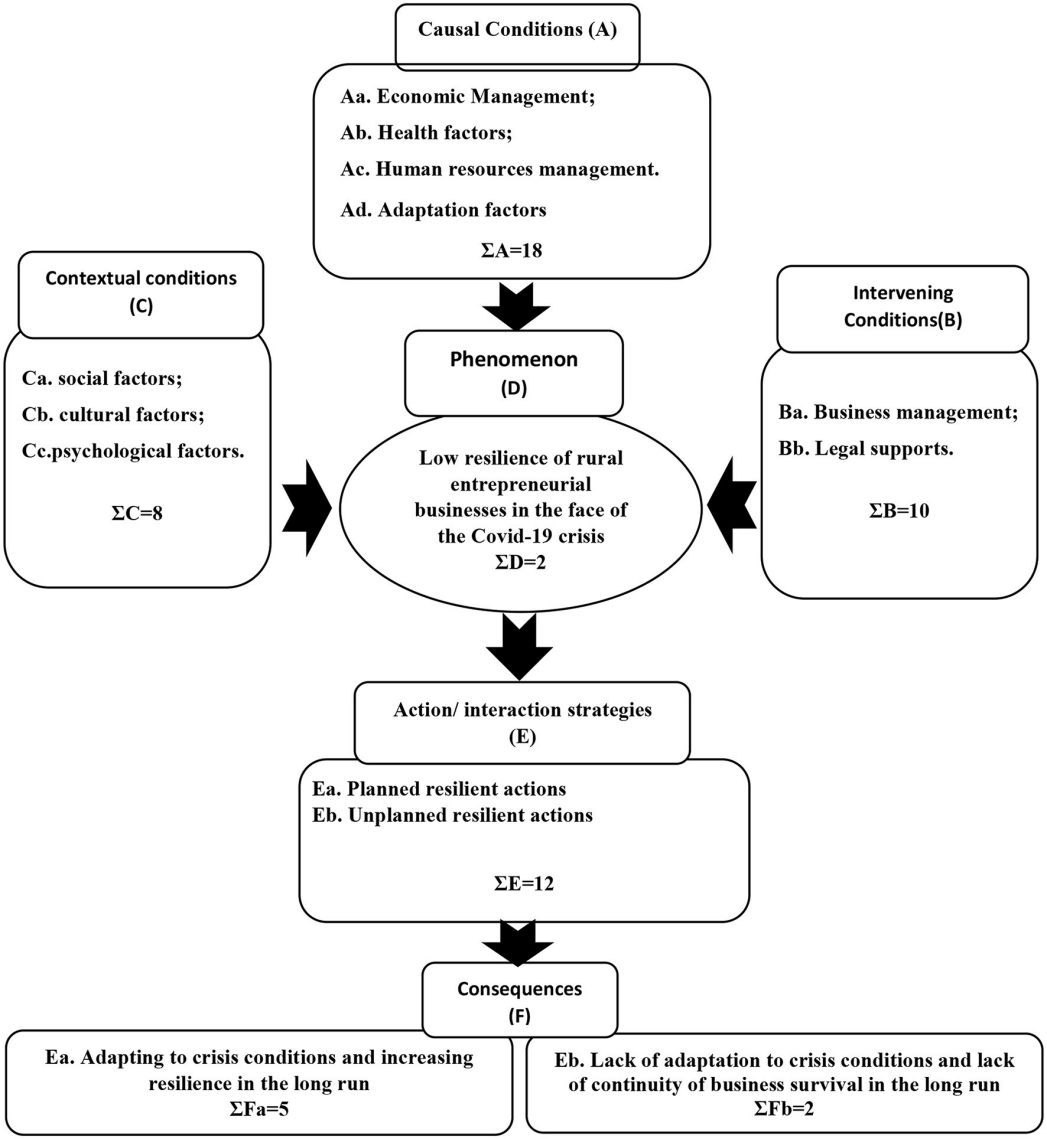
Paradigm model of the resilience of rural entrepreneurial businesses in the face of the COVID-19 crisis.

Figure 3 shows the paradigm model of the resilience of rural entrepreneurial businesses in the face of the COVID-19 crisis.

### Selective Coding

The purpose of selective coding is to identify the main phenomenon and relate it logically to the different subcategories through the paradigm model. To do this, the researcher uses a storyline to describe and justify the relationships between the categories and presents a visual model of the main research topic (34).

There are different criteria for judging which category should be considered the main category. (55) provides a list of criteria that can be used to determine whether a category is eligible to be selected as the main category:

1. This category should be the main (pivotal) category. That is, it can be related to many other categories and their characteristics, but is more suitable for the main category than other candidates. This criterion of centrality is a necessary condition for placing a category at the heart (center) of the analysis: it indicates that this category describes a major part of the changes in the pattern of behavior.
2. The main category must be observed repeatedly in the data. With high repetition, this category is considered as a stable pattern and is consequently related to other categories by the analyst.
3. The main category is easily related to other categories. These communications are not mandatory; rather, they come into being quickly and abundantly. Because the main category is related to many other categories and is repeated frequently, it becomes more saturated in more time than the other categories.
4. The main category in a substantive study has clear implications for a more general theory.
5. Because the details of a main category are generated analytically, the theory moves forward appreciably.

As mentioned earlier, the research phenomena included two subcategories (Da1 and Da2). According to Strauss (1987) criteria, the most important phenomenon in this research involved the Da1 subcategory. This subcategory is referenced 26 times.

As shown in Figure 3, the main phenomenon is caused by four subcategories including economic management, health factors, human resources management, and adaptation factors. These four subcategories include a total of 18 concepts. Economic management was referenced a total of 40 times by the interviewees, which included six concepts (References mean: 6.66). These six concepts include receiving disaster loans (crisis), income diversity, previous financial resources, liquidity management, control and reduce costs, manage and increase sales. Among these concepts, the most important concept was “Liquidity management” with nine references (most references). In other words, in the economic management sub-category, the “Liquidity management” factor has a greater impact on increasing the resilience of rural entrepreneurial businesses during the crisis. Health factors were referenced a total of 37 times by the interviewees, which included five concepts (References mean: 7.4). These five concepts include equipping the business environment with various sanitary devices (proper ventilation, etc.), providing disposable health tools to personnel in order to comply with protocols (masks, etc.), equipping businesses with early diagnostic tools (Fever gauge, etc.), adherence to health protocols, and daily staff checkup. Among these concepts, the most important concept was “Adherence to health protocols” with 10 references (most references). In other words, in the health factors sub-category, the “Adherence to health protocols” factor has a greater impact on increasing the resilience of rural entrepreneurial businesses during the crisis. Human resources management was referenced a total of 35 times by the interviewees, which included four concepts (References mean: 8.75). Among these concepts, the most important concept was “Improving the skills of personnel in observing health protocols” with 10 references (most references). In other words, in the human resources management sub-category, the “Adherence to health protocols” factor has a greater impact on increasing the resilience of rural entrepreneurial businesses during the crisis. Adaptation factors were referenced a total of 26 times by the interviewees, which included three concepts (References mean: 8.66). Among these concepts, the most important concept was “Use of crisis adaptive technologies in business model” with 11 references (most references). In other words, in the adaptation factors sub-category, the “Use of crisis adaptive technologies in business model” factor has a greater impact on increasing the resilience of rural entrepreneurial businesses during the crisis. In other words, the factors that directly increase the resilience of rural entrepreneurial businesses include four factors: economic management, health factors, human resource management and adaptation factors. Therefore, rural entrepreneurial business managers seeking resilience should consider these four factors in their planning during a crisis. In general, these four subcategories (Causal Conditions) directly affected the occurrence of the phenomenon and indirectly (through the phenomenon) affected the action / interaction strategies (Figure 3).

According to the participants, in addition to causal conditions, the contextual and intervening conditions also affect the phenomenon (Figure 3).

Contextual conditions include three subcategories: social factors, cultural factors and psychological factors. These three subcategories include a total of eight concepts. Social factors were referenced a total of 15 times by the interviewees, which included three concepts (References mean: 5). Among these concepts, the most important concept was “Increasing trust, participation and cooperation in observing preventive measures among personnel and customers” with six references (most references). In other words, in the social factors sub-category, the “Increasing trust, participation and cooperation in observing preventive measures among personnel and customers” factor has a greater impact on increasing the resilience of rural entrepreneurial businesses during the crisis. cultural factors were referenced a total of 14 times by the interviewees, which included two concepts (References mean: 7). Among these concepts, the most important concept was “Strengthening the entrepreneurial culture in business with the aim of providing products or services that are creative and adapt to new conditions and gain a competitive advantage” with eight references (most references). In other words, in the cultural factors sub-category, the “Strengthening the entrepreneurial culture in business with the aim of providing products or services that are creative and adapt to new conditions and gain a competitive advantage” factor has a greater impact on increasing the resilience of rural entrepreneurial businesses during the crisis. Psychological factors were referenced a total of 21 times by the interviewees, which included three concepts (References mean: 7). Among these concepts, the most important concept was “Increase staff resilience in relation to business continuity” with eight references (most references). In other words, in the psychological factors sub-category, the “Increase staff resilience in relation to business continuity” factor has a greater impact on increasing the resilience of rural entrepreneurial businesses during the crisis. In general, contextual conditions provided the context for the phenomenon to occur in the study population. Social factors, cultural factors and psychological factors were subcategories that formed contextual conditions. According to the participants, these three sub-categories (contextual conditions) directly affected the occurrence of the phenomenon and indirectly (through the phenomenon) affected the action / interaction strategies (Figure 3).

Intervening conditions include two subcategories: Business management and Legal supports. These two subcategories include a total of 10 concepts. Business management was referenced a total of 24 times by the interviewees, which included three concepts (References mean: 8). These three concepts include crisis management and proper accountability, condition-based planning for the business, develop and present appropriate strategies for business continuity. Among these concepts, the most important concept was “develop and present appropriate strategies for business continuity” with nine references (most references). In other words, in the Business management sub-category, the “develop and present appropriate strategies for business continuity” factor has a greater impact on increasing the resilience of rural entrepreneurial businesses during the crisis. Legal supports were referenced a total of 59 times by the interviewees, which included seven concepts (References mean: 8.42). Among these concepts, the most important concept was “Proper management and policy making” with 12 references (most references). In other words, in the Legal supports sub-category, the “Proper management and policy making” factor has a greater impact on increasing the resilience of rural entrepreneurial businesses during the crisis. As mentioned earlier, the two subcategories of Business management and Legal support were intervening conditions that also facilitated the occurrence of the phenomenon. In general, according to the participants, these two sub-categories (intervening conditions) directly affected the occurrence of the phenomenon and indirectly (through the phenomenon) affected the action / interaction strategies (Figure 3).

When the phenomenon occurred, the study population took action to cope with the phenomenon. In this study, according to the participants, the measures taken were classified into two subcategories, planned resilient actions and unplanned resilient actions. These two subcategories include a total of 12 concepts. Planned resilient actions were referenced a total of 54 times by the interviewees, which included seven concepts (References mean: 7.71). Among these concepts, the most important concept was “Development and modification of marketing strategies based on crisis conditions” with 10 references (most references). Unplanned resilient actions were referenced a total of 25 times by the interviewees, which included five concepts (References mean: 5). Among these concepts, the most important concept was “Temporary deactivation of the business” with six references (most references). According to the participants, these two sub-measures had two sub-categories in consequences, which are discussed below.

Finally, according to the participants, implementing action/interaction strategies can have both positive (Fa) and negative consequences (Fb). The consequences of planned resilient actions (positive consequences), while adapting to crisis conditions, provide business growth and development in the long run. The sub-category adapting to crisis conditions and increasing resilience in the long run had five concepts that were referenced 38 times (References mean: 7.6). In the meantime, the concept of “Improvement of business position than before crisis” with nine references had the most references, in other words, this was the most important positive consequence. Negative consequences included unplanned actions that in the short term may have led to adaptation to the crisis, but in the long term would have led to the bankruptcy and destruction of the business. This subcategory had two concepts that had 13 references in total (References mean: 6.5). Meanwhile, the concept of “More vulnerability in the long run” with seven references had the most references, in other words, this was the most important negative consequence. In general, according to the participants, some action strategies such as planned resilient actions can help the resilience of rural entrepreneurial businesses both in the short term and in the long term. If resilient actions are planned, it will lead to business growth and development in both the long and short term. Otherwise, the actions may be resilient in the short term, but in the long run, they will cause business bankruptcy will be. Based on this discussion, the final conceptual model of the research is presented in Figure 3.

## Conclusions and Recommendations

The purpose of this study is to develop a paradigm model for resilience of rural entrepreneurial businesses in dealing with the COVID-19 crisis with application of grounded theory. The outbreak of COVID-19 led to disruption to businesses, especially rural entrepreneurial businesses. Therefore, rural entrepreneurial businesses need to adapt to change in order to create the desired changes to build a better future in order to survive. Therefore, it is necessary to make systematic plans to increase resilience and use planned resilience strategies. Resilience creates capabilities for businesses that can adapt to adverse conditions and continue to survive and return to development. Resilience helps the long-term survival of rural entrepreneurial businesses. As a result, these businesses can continue to operate by using the dimensions of resilience strategies in the context of the COVID-19 crisis, increasing their resistance to the Corona crisis and overcoming the crisis more developed than before. What should especially be addressed to rural entrepreneurial business managers is that adaptive measures should be taken in the long run in the direction of business development and growth, and any tolerant retaliatory action should be avoided.

Grounded theory is a quite powerful tool based on which the factors affecting the phenomenon (the main problem) can be investigated. Therefore, based on this, appropriate recommendations can be provided to solve the problem. Although the infrastructure is currently weak in most villages, the next step is to strengthen the infrastructure, especially in the field of information and communication technology in rural areas. The next step is to upgrade the IT skills of managers and business personnel. Strengthening these infrastructures and upgrade the IT skills of managers and business personnel in rural areas gives rural entrepreneurial businesses the opportunity to redesign their business model based on information technology. The most important consequence is the adaptation of rural entrepreneurial businesses to the COVID-19 crisis and also, the development of information technology infrastructure has led to the development of marketing for the sale of products worldwide. Market development helps both economic management and reducing customer interactions (Reducing customer interactions means observing health factors and proper human resource management). In other words, by strengthening the infrastructure and developing skills, the context for the use of information technology in rural entrepreneurial businesses is provided, and this means the realization of all four subcategories of causal conditions. Social factors, cultural factors and psychological factors should also be strengthened through cyberspace in order to provide a suitable platform for dealing with the COVID-19 crisis.

Grounded theory is a quite powerful tool also because it examines the actions taken as well as their positive and negative consequences one by one. Therefore, with proper planning, negative actions and consequences can be eliminated or reviewed, and positive actions and consequences can be strengthened and expanded. Investigation of the measures taken and their consequences showed that rural entrepreneurial business managers should have a long-term vision for the continuation of their business. In this regard, strategies should be adopted that, while increasing adaptation in the short term, also lead to the growth and development of the business in the long term.

Based on the findings, the following suggestions are made;

- It is recommended that the Continuous data collection, analysis and presentation of information and market trends consumed in the current situation and the coming years with the aim of strengthening futurology and identifying and exploiting future opportunities to gain a competitive advantage;
- Recommends that the level of creative thinking of managers be strengthened with the aim of increasing the ability to adapt to critical situations;
- Recommends that managers' strategic planning skills be strengthened, especially in the field of vision drawing and mission determination;
- It is recommended that the government provide loans and disaster subsidies, as well as business managers to manage cash flow and reduce unnecessary costs;
- It is recommended that the relevant organizations try to increase and update the skills and knowledge of business personnel in how to deal with and adapt to health by holding training classes (mostly virtual);
- It is recommended that business owners provide a safe environment for staff and provide free sanitary equipment to staff in order to protect human capital;
- It is recommended that the IT infrastructure in the villages be strengthened so that businesses can use it to redesign their business model;
- It is recommended that the field of increasing virtual communication between business owners and relevant organizations in the region and the province be maintained and strengthened to share crisis adaptation experiences;
- It is recommended that resilient measures be taken systematically and in a long-term perspective in order to increase resilience during the crisis and lead to long-term business growth and development;

It is also recommended that the findings of this study be made available to rural business managers through training programs (mainly in cyberspace).For future research, it is proposed to provide a model of adaptation behavior of rural entrepreneurial businesses in the face of the COVID-19 crisis. The most important limitation of the present study can be expressed as the lack of cooperation of some of the study population due to fear of COVID-19.

## Data Availability Statement

The raw data supporting the conclusions of this article will be made available by the authors, without undue reservation.

## Author Contributions

EK and FK wrote the first draft of the manuscript. NN contributed in data collection. All authors contributed to conception and design of the study and performed the statistical analysis, wrote sections of the manuscript, contributed to manuscript revision, read, and approved the submitted version.

## Conflict of Interest

The authors declare that the research was conducted in the absence of any commercial or financial relationships that could be construed as a potential conflict of interest.

## Publisher's Note

All claims expressed in this article are solely those of the authors and do not necessarily represent those of their affiliated organizations, or those of the publisher, the editors and the reviewers. Any product that may be evaluated in this article, or claim that may be made by its manufacturer, is not guaranteed or endorsed by the publisher.
